# Dilation of the superior sagittal sinus detected in rat model of mild traumatic brain injury using 1 T magnetic resonance imaging

**DOI:** 10.3389/fneur.2023.1045695

**Published:** 2023-04-26

**Authors:** Jennie M. Burns, Benjamin T. Kalinosky, Mark A. Sloan, Cesario Z. Cerna, David A. Fines, Christopher M. Valdez, William B. Voorhees

**Affiliations:** ^1^General Dynamics Information Technology, Defense Division, JBSA Fort SamHouston, TX, United States; ^2^Radio Frequency Bioeffects Branch, Bioeffects Division, Airman Systems Directorate, 711th Human Performance Wing, Air Force Research Laboratory, JBSA FortSam Houston, TX, United States

**Keywords:** brain, low field, MRI, rat, TBI, vasodilation, vasculature

## Abstract

**Introduction:**

Mild traumatic brain injury (mTBI) is a common injury that can lead to temporary and, in some cases, life-long disability. Magnetic resonance imaging (MRI) is widely used to diagnose and study brain injuries and diseases, yet mTBI remains notoriously difficult to detect in structural MRI. mTBI is thought to be caused by microstructural or physiological changes in the function of the brain that cannot be adequately captured in structural imaging of the gray and white matter. However, structural MRIs may be useful in detecting significant changes in the cerebral vascular system (e.g., the blood-brain barrier (BBB), major blood vessels, and sinuses) and the ventricular system, and these changes may even be detectable in images taken by low magnetic field strength MRI scanners (<1.5T).

**Methods:**

In this study, we induced a model of mTBI in the anesthetized rat animal model using a commonly used linear acceleration drop-weight technique. Using a 1T MRI scanner, the brain of the rat was imaged, without and with contrast, before and after mTBI on post-injury days 1, 2, 7, and 14 (i.e., P1, P2, P7, and P14).

**Results:**

Voxel-based analyses of MRIs showed time-dependent, statistically significant T2-weighted signal hypointensities in the superior sagittal sinus (SSS) and hyperintensities of the gadolinium-enhanced T1-weighted signal in the superior subarachnoid space (SA) and blood vessels near the dorsal third ventricle. These results showed a widening, or vasodilation, of the SSS on P1 and of the SA on P1–2 on the dorsal surface of the cortex near the site of the drop-weight impact. The results also showed vasodilation of vasculature near the dorsal third ventricle and basal forebrain on P1–7.

**Discussion:**

Vasodilation of the SSS and SA near the site of impact could be explained by the direct mechanical injury resulting in local changes in tissue function, oxygenation, inflammation, and blood flow dynamics. Our results agreed with literature and show that the 1T MRI scanner performs at a level comparable to higher field strength scanners for this type of research.

## Introduction

In the United States, traumatic brain injury (TBI) in humans is a common injury with a recent report of approximately 2.9 million diagnoses in 2014 (1). TBI is caused by an external force applied to the head or body in a manner resulting in the disruption of normal brain function. The severity of TBI is classified as mild, moderate, and severe and can be diagnosed based on the duration of loss of consciousness, coma rating scale, post-traumatic amnesia, and brain imaging results (2). Mild TBI (mTBI) is estimated to consist of 70–90% of treated TBI cases (3). The onset of mTBI typically results in a brief period of unconsciousness (<30 min) and may be followed by impaired motor movement, balance, and cognitive function (such as learning, memory, concentration, and problem solving). Other symptoms may also include headaches, fatigue, depression, anxiety, and irritability and often resolve within 3 months post-injury. However, for individuals with post-concussion syndrome, these collective disabilities can be life-long and may inhibit them from rejoining regular work, social, and family life (4, 5).

Magnetic resonance imaging (MRI) is a clinical tool widely used to diagnose and study brain injuries and diseases; however, mTBI remains notoriously difficult to detect with structural MR imaging (i.e., T1-weighted and T2-weighted imaging). mTBI is caused by microstructural or physiological changes in the function of the brain that cannot be adequately captured in gross structural imaging of the gray and white matter, which explains the insensitivity of the modality in detecting mTBI (5–7). However, structural and contrast-enhanced MRI may be useful in detecting other significant changes in the non-gray and white matter areas of the brain that can have an impact on brain function, such as the cerebral vascular system and the ventricular system. Under normal physiological conditions, cerebral blood flow (CBF) is autoregulated to be constant in response to variations in systemic blood pressure via vasodilation and vasoconstriction, primarily in the cerebral arteriolar and capillary beds (8, 9). Following mTBI, CBF is known to change but there are conflicting observations of CBF increasing and decreasing at different time points post injury (9). The primary objective of this study was to use a 1 T permanent magnet MR system to take structural and contrast-enhanced images of the brain of a rat after mTBI and determine if changes in the white and gray matter of the brain and blood–brain barrier (BBB) could be detected. Additionally, if changes in the brain matter are detected, could the changes be indicative of changes in the cerebrovascular and ventricular systems? Understanding these effects is important in expanding our knowledge of the pathophysiology of mTBI and could lead to better methods to identify and treat these injuries and alleviate the symptoms in humans.

Another objective was to determine if this study could be accomplished using a permanent magnet 1 T preclinical MRI scanner, which is considered to have relatively low magnetic field strength compared to modern clinical-grade MRIs (> > 1.5 T). Although MRI scanners with magnetic field strengths ≤1 T in general have several disadvantages compared to the use of higher field strength MRIs (such as long scan times and an inability to perform certain types of imaging techniques), these MRIs also have several technical and financial advantages. Low-field MRI scanners are usually more affordable in terms of cost, maintenance, power consumption, required operator manpower (particularly because it is safe and easy for novices or students to operate), and sustainability. They are less complex (e.g., permanent magnets may be used instead of superconducting magnets), have a much smaller footprint allowing use in laboratories with limited space (e.g., in laboratories where space is nearly fully utilized or in laboratories with higher containment level), and can be portable, increasing accessibility (10). These MRIs can also be configured to perform clinical standard scans, test new scans, and, in some instances, lower field strengths can perform better imaging (10, 11). These advantages make low-field MRI scanners attractive for small research laboratories or clinics operating on small or tight budgets, particularly in resource-poor settings, or for laboratories conducting preclinical or preliminary/startup research.

Many techniques have been used by researchers to emulate, with controlled conditions, human mTBI in the rat animal model (12). In this study, mTBI was emulated in the anesthetized rat using the “Marmarou” linear acceleration drop-weight technique, a technique commonly used to induce a range of TBI severities. To induce an mTBI, a 450 g weight was dropped from a height of 1 m onto the skull of a rat. For rat-to-human impact comparisons, Viano, Hamberger (13) used the Marmarou technique in rats and dropped a 450 g weight from a height of 1 m and measured a peak head acceleration of 603 ± 187 × *g*. This mean peak head acceleration in the rat was scaled to be approximately equivalent to a mean peak head acceleration reported in the human of 69.6 ± 23.6 × *g* (4) and maximum translational acceleration at the center of gravity of the human head of 66 × *g* corresponding to a 25% probability of mTBI (14). In Viano, Hamberger (13), these injuries in the rat corresponded to a <20% probability of brain injury. Therefore, we expected <20–25% probability of inducing an mTBI in our animal model and, if any injury had occurred, the injury would be difficult to detect in structural MRI. For comparison, in clinical cases of mTBI, abnormalities in the gray and white matter of the brain are usually undetectable in structural MRI scans and other abnormalities, such as subdural or subarachnoid hemorrhage, are found in only 30% of those scans (6). Since our work was a 14-day post-injury survival study, this “Marmarou” drop-weight impact was chosen based on its common use and categorization as a model of mTBI, its low risk of skull fracture, its low morbidity and mortality rates, and its non-zero probability of showing subtle, yet significant, brain injury in MRI and histology (13).

## Materials and methods

### Animals and injuries

#### Animal use and care

The animals involved in this study were procured, maintained, and used according to an Institutional Animal Care and Use Committee (IACUC)-approved Animal Use Protocol and established animal welfare standards, compliant with: DoD Instruction 3216.01 (15); U.S. Department of Agriculture Animal Welfare Regulations (16); the Guide for the Care and Use of Laboratory Animals, 8th Edition, National Research Council (17); and DHA-MSR 6025.02 (18). The Air Force Research Laboratory at Joint Base San Antonio (JBSA) Fort Sam Houston, Texas has been accredited by AAALAC International since 1967.

Ten (*n* = 10) male, Sprague Dawley rats (*Rattus norvegicus*; CD rats, Charles River Laboratories, Wilmington, MA) weighing 298 to 340 g (315 ± 10 g, mean ± SD of measurements taken on each day of MRI and on the day of the injury) were used in this study. Animals were ordered at a weight ≤ 200 g and were group housed upon arrival. Upon arrival, animals were allowed *ad libitum* access to water and standard rat chow until their weight was above 300 g, at which point the animals were single-housed and allowed *ad libitum* access to water while food was regulated to maintain their weight (i.e., each animal was given at least 12 g or 2–3 pellets of standard rat chow each day). Animals were single-housed in order to control food consumption for maintaining animal weight within required limits. Animal bodyweights were regulated to be within 300 to 350 g (±5% error on maximum limit) for applying consistent injuries (i.e., head and brain size matters with regard to the mechanical dynamics of the brain during impact); weight restriction also allowed the animals to be below the bodyweight limit of the MRI head coil/carrier, with an approximate rat bodyweight limit of 350 g, during the 20-day time course of the study. Bodyweight was monitored 5 days a week by veterinary personnel. The animal weights recorded on each day prior to the MRI scan are shown in Table 1. Note that in Table 1, animal bodyweights on days B1 and B2 were averaged and reported as B, and that *n_s_* = number of samples used to calculate the statistics in Table 1. The animals were provided rat-appropriate environmental enrichment (e.g., huts and nesting material) throughout the study.

**Table 1:** Animal bodyweight (g) and age (d) on day of MRI and drop-weight injury.

	B	Injury	P1	P2	P7	P14	TOTAL
Mean	308 g (65 d)	310 g (68 d)	322 g (69 d)	317 g (70 d)	319 g (75 d)	325 g (82 d)	315 g (71 d)
SD	6 g (3 d)	5 g (4 d)	5 g (4 d)	8 g (4 d)	7 g (4 d)	9 g (4 d)	10 g (7 d)
*n_s_*	20	10	10	10	10	10	70

#### Drop-weight procedures

All subjects in this study were anesthetized and underwent a closed-head, linear acceleration, drop-weight procedure to induce an mTBI. The technical methodology for inducing mTBI followed that of Marmarou, Foda (19). In a surgical suite, the animal was anesthetized with vaporized isoflurane (2–5% for induction with O_2_) within an induction chamber. The subject was then transferred to a stereotaxic frame and placed on a heating pad to maintain body temperature. The subject was positioned within the nose cone of the frame to continue administering isoflurane (2–5% isoflurane with O_2_ at a flow rate of 2–4  l/min) to achieve a surgical plane of anesthesia (i.e., no reflex responses to tail or toe pinch). Isoflurane was administered throughout surgery. The external meatus of the ears were coated in topical, 2% lidocaine hydrochloride jelly and the ear bars of the stereotaxic frame were placed into the external meatuses and fastened to the frame. Rectal body temperature, respiration rate, heart rate, and blood oxygenation were monitored. The subject was administered a perioperative dose of cefazolin (5–10 mg/kg, subcutaneous (SC)) and buprenorphine SR Lab (1.2 mg/kg, SC) 30 min prior to surgical incision (20). The scalp was then shaved and the surgical site (i.e., the skin on the superior surface of the cranium) was thoroughly cleaned using sterile surgical scrub solution, 70% isopropyl alcohol, and gauze sponges. SC injections of local anesthetic agent (1% lidocaine, 0.05–0.1 ml) were administered to the surgical site and the area was again thoroughly cleaned. After confirming the animal was in the surgical plane of anesthesia, a midsagittal incision was made in the skin of the surgical site. The skin and periosteum were reflected to expose the bone sutures (midsagittal suture, bregma, and lambda) of the skull. Sterile gauze pads were used to keep the area dry. A clean, sterilized, stainless-steel disc [10 mm in diameter, 3 mm thick, and custom-made based on specifications in Marmarou, Foda (19)] was fixed to the skull between bregma and lambda on the midsagittal suture, such that the rostral edge of the disc was tangent with bregma, using temporary dental adhesive (Fast curing denture repair acrylic resin powder and liquid, REF 1223, Jet™ Denture Repair Package; Lang Dental Mfg. Co., Inc., Wheeling, IL).

The drop-weight apparatus was custom-made and used according to the diagram and specifications in (19, 21, 22). The apparatus consisted of a long vertical plastic tube, in which a metal weight could be dropped vertically from a controlled height, attached to a stand suspended above an acrylic box containing the specified, low-density polyurethane, foam bed (Type E bed, Foam to Size Company, Ashland, VA) of known mechanical properties (23). The foam bed was covered with a thin, loosely laid, absorbent cotton pad for clean reuse of the bed between subjects.

After the adhesive dried (~20 min), the subject was quickly removed from the stereotaxic and nose cone and placed on the foam bed of the drop-weight apparatus. The disc on the head of the subject was aligned with the bottom of the drop-weight tube and the 450 g weight was dropped from a height of 1 m to induce mTBI. After the weight dropped and rebounded, a slide door at the bottom of the tube was closed to trap the weight inside the tube in order to prevent additional impacts. The subject was then immediately placed back into the nose cone of the stereotaxic frame for continued isoflurane administration. The disc and adhesive were removed from the skull, and the incision in the skin was closed using skin adhesive (Dermabond® Advanced Topical Skin Adhesive; Ethicon, Inc., Raritan, NJ). For recovery, the subject was then taken off the isoflurane and placed in a clean cage with a heating pad to maintain body temperature. After the subject recovered from anesthesia, the subject was placed back in its home cage and underwent a 72 h period of post-surgical/injury observation. In the home cages, the enrichment huts consisting of hard plastic were replaced with paper huts to prevent additional potential injury to the head.

#### Post-surgical care and monitoring

Post-surgical and injury procedure, the animals were monitored until fully recovered from anesthesia. One subject displayed apnea with onset immediately following the drop-weight procedure and breathing was assisted until the subject was capable of unassisted breathing. The spO_2_ remained within normal range throughout the intervention and the subject made a full recovery with no post-procedural behavioral abnormalities. No other post-procedural interventions were necessary for any animals. All animals were given cefazolin (5–10 mg/kg, SC) and monitored bi-daily for 72 h using a rat pain/distress assessment score sheet. Scores were allowed to range from 0 to 3 on various assessments of the appearance, behavior, and potential injury. The highest score, with a score > 1, in any of the categories dictated the level of intervention (e.g., additional doses of buprenorphine SR Lab) and additional number of observations as determined by the veterinarians. If the score remained high at the 72 h post-surgical/injury procedure time point, scoring would continue a minimum of 1x daily until the animal reached a score of 0–1. However, no subjects in this study received scores >1 and scoring did not continue past the 72 h observation time point.

#### Euthanasia

After the study was complete on P14, the animals were deeply anesthetized via 4% isoflurane inhalation into a surgical plane of anesthesia to administer euthanasia. Euthanasia was administered by overdose (1 ml intracardiac injection) of pentobarbital sodium and phenytoin sodium (Euthasol® Euthanasia Solution; 100 mg/kg bodyweight, 390 mg/ml concentration) and was confirmed by the absence of vital signs.

### MRI procedures and protocols

MRI was performed on a 1 T permanent magnet scanner (M7 system, Aspect Imaging, Shoham, Israel). Prior to imaging, the scanner underwent frequency, coil, shim, and radiofrequency calibration. All subjects were anesthetized and underwent imaging on two separate baseline days, during the period of 1–5 days prior to injury (i.e., B1 and B2), and on post-injury days 1, 2, 7, and 14 (i.e., P1, P2, P7, and P14); the MRI schedule is shown in Table 2.

**Table 2:** MRI schedule (days).*

-5	-4	-3	-2	-1	0	1	2					7							14

* Drop-weight injury was on day 0 (black box) with all gray boxes indicating days of MRI; however, note that subjects were imaged on just two of the 5 days preceding injury.

Each rat was sedated using 2–4% isoflurane inhalation in an induction chamber and then placed on a benchtop heating pad and nose cone for continued administration of isoflurane in order to place a catheter in the lateral tail vein (22 G or smaller). The animal was then placed in the prone position in the head coil of the rat carrier with an integrated face-cone and bite-bar for continuous anesthesia by inhalation (1–3% isoflurane with O_2_ at a flow rate of 2–4  l/min). An integrated heating pad was used for maintaining normal body temperature during MRI acquisition. We placed physiological sensors on the rat for continuous monitoring and recording of the core body temperature (via a rectal thermometer), respiration rate (via a pneumatically controlled pressure pad placed beneath the abdomen of the rat), and heart rate and blood oxygen saturation (via a pulse oximeter placed on a hind-limb paw) throughout imaging (Model 1030 MR-compatible Monitoring and Gating System for Small Animals, Small Animal Instruments, Inc., Stony Brook, NY). The rat in the head coil/carrier was then slid into place within the magnet of the scanner. Fast scout scans with preset region of interest (ROI) markers were used to carefully align the subject in the scanner for consistent slice positioning between subjects and days of imaging. The subject was then imaged using the protocol scans and parameters shown in Table 3.

**Table 3:** MRI scans and parameters.

	T2W FSE Sagittal and Axial	T1W SE Sagittal and Axial	T1W 3D GRE Axial
TR (ms)	4,000	600	27
TE (ms)	67.7	15.0	6.4
Flip Angle (^o^)	-	-	30
FOV (mm)	35 × 35	35 × 35	35 × 35
Acquisition Matrix (# samples × # phase encodings)	168 × 168	168 × 168	175 × 175
Resolution (mm)	0.208 × 0.208	0.208 × 0.208	0.200 × 0.200
Pixel Spacing (mm)	0.137 × 0.137	0.137 × 0.137	0.137 × 0.137
Slice gap (mm)	1.5	1.5	1.0
Slice thickness (mm)	1.0	1.0	1.0
Number of Slices	17	17	28
NEX	20	10	3
Duration (min:sec)	21:20	18:48	6:54

All subjects underwent two T1W GRE axial imaging scans (one pre- and one post-contrast enhancement) for the last two scans of the protocol such that the post-contrast scan was conducted after an intravenous bolus injection of gadoteridol followed by a saline flush via the tail-vein catheter. Pharmaceutical-grade gadoteridol (1.2 mg/kg bodyweight, 279.3 mg/ml concentration; ProHance®, Bracco Diagnostic Inc., Monroe Township, NJ) was injected at a rate of 3 ml/min followed by an injection of 0.9% physiological saline (0.24 ml) at the same rate of 3 ml/min (24) via a syringe pump (MRI compatible syringe pump, Harvard Apparatus, Holliston, MA). This gadoteridol dose corresponds to a clinical dose of 0.1 mmol/kg bodyweight in humans and is based on extrapolation to rats described in the US Food and Drug Administration (FDA) guidance for Human Equivalent Dose (25). After the contrast-enhanced MRI was complete, the subject was taken out of the scanner and the tail-vein catheter was removed. For recovery, the subject was placed on a benchtop heating pad and administered O_2_ (inhalation at a flow rate of 2–4  l/min) via a nose cone and the vitals were monitored until fully recovered.

### MR image processing pipeline and statistical analyses

**Figure 1:**
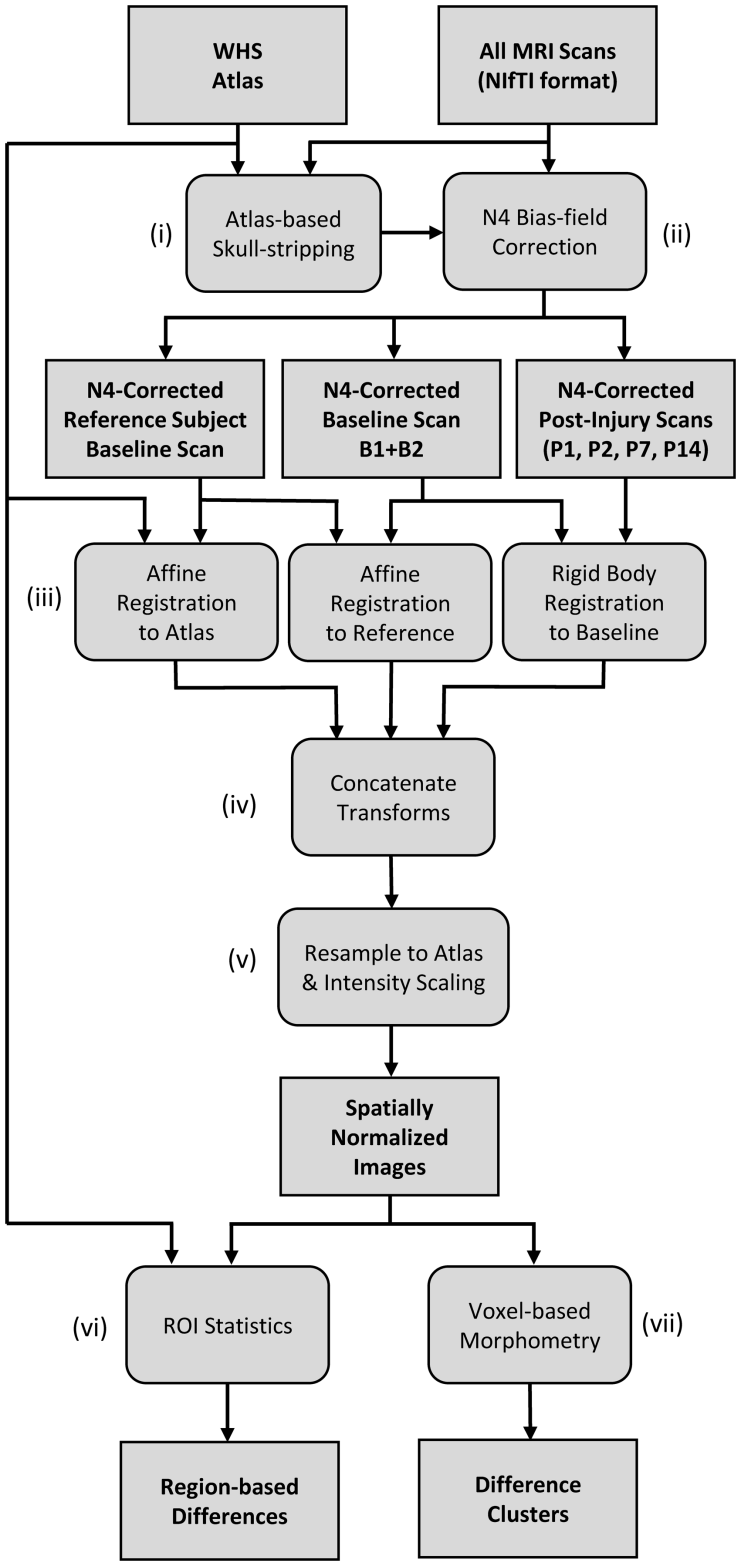
Flow-chart of the MR image processing pipeline for region- and voxel-based comparisons.

A flowchart of the MR image-processing pipeline is shown in Figure 1. The Waxholm Space (WHS) rat brain atlas was used for the registration of all MR images in this study. The WHS rat brain atlas is an open access, volumetric, brain atlas with segmentations of the Sprague Dawley rat brain (80 days old, male) described in detail in (26–28), provided through an online database (29), and viewable online (30). The atlas is an *ex vivo*, T2* weighted volumetric image with an isotropic resolution of 39 μm and 80 segmented brain regions [including regions of white matter, gray matter, and cerebral spinal fluid (CSF)]. The rat brain atlas was down-sampled to match the in-plane resolution of the anisotropic MRI scan being analyzed, and resliced to match the anatomical plane of the scan analyzed (i.e., in-plane axial or sagittal).

The image-processing pipeline and group analyses were developed using a collection of in-house tools and scripting software. In-house tools were written in Python (v. 3.8) and Java (v. 17), and made use of the Insight Toolkit (ITK; v.5.1) (31, 32) and SimpleITK (v.5.1) (33, 34). ImageJ/FIJI (v.1.53c) (35) software was used for data visualization during development. Prior to image processing, the DICOM file formatted image sequence of each scan was initially converted into a 3D, volumetric MR image in NIfTI file format. As indicated in Figure 1i, an atlas-guided skull-stripping routine was used to segment the brain in each individual scan. Each MRI scan underwent initial affine registration with the brain mask of the WHS atlas using the gradient descent optimization algorithm and a correlation objective function. The inverse transform was used to map the atlas brain mask into the subject space to produce individualized brain masks. Following skull-stripping, each scan and its brain mask were provided to the N4ITK program for bias field correction (36) as indicated in Figure 1ii.

The bias field-corrected images were spatially normalized with a set of rigid body and affine registrations (Figure 1iii). Each subject’s baseline image (B) underwent an affine registration with a chosen reference subject baseline day scan. We chose baseline scan B1 of an arbitrary subject as our reference scan. The reference scan was then aligned to the WHS rat brain atlas using a separate affine registration. All post-injury images (P1, P2, P7, and P14) of each subject were coregistered to the same subject’s baseline (B) image using rigid body registration. As shown in Figure 1iv, a composite transform was produced by concatenating the intra-subject rigid body transforms with the two affine transforms that first map to the reference subject coordinate system and then to the Waxholm atlas space. Finally, the inverse of the composite transform was used to spatially normalize each scan to the common stereotaxic Waxholm atlas space (Figure 1v). The spatially normalized brains were also rescaled in intensity to be equal in mean value within the masked brain region. This rescaling was done to correct for observed variations in global signal intensity between scan sessions. The spatially normalized images were resampled to an isotropic resolution of 0.1367 mm to match the in-plane resolution of the T2W FSE and T1W SE scans.

After spatially normalizing all images to the Waxholm atlas space, both region-based (Figure 1vi) and voxel-based (Figure 1vii) analyses were used to compare groups. The spatially normalized images were grouped by the day of scan; however, all baseline images (B1 and B2) were grouped together (designated as B). Baseline images were compared to each post-injury day scan for each MRI scan type [T1W SE, T2W FSE, and T1W GRE+ Gadolinium (Gd) images]. We also performed group comparisons on the pre- to post-Gd contrast-enhanced T1W GRE images for each scan session. The pre-contrast image was subtracted from the rigidly aligned post-contrast image at each voxel to produce a Gd-difference image. The number of datasets included in the analyses are listed in Table 4; some images were excluded from the analyses due to user error (i.e., incorrect settings), system error, noise, or artifacts.

**Table 4:** Number of datasets used in statistical comparisons by MRI scan and day.

Day	T2W	T1W		T1W GRE	T1W GRE + Gd	T1W GRE + Gd Difference
	Sagittal and Axial	Sagittal	Axial	Axial (pre-Gd)	Axial (post-Gd)	Axial (difference)
B	20	20	19	18	18	18
P1	10	10	10	10	10	10
P2	10	10	9	8	9	8
P7	10	10	10	10	10	10
P14	10	10	10	9	10	9

For region-based analyses, the mean image value was calculated in each of the 80 regions from the WHS atlas. Unpaired, two-tailed Student’s t-tests were performed between the baseline group and each post-injury day group. (Note that unpaired t-tests were used instead of paired t-tests to account for the additional variability induced in each scan by a combination of the interslice gaps and field of view (FOV) positioning between sessions.) A false-discovery rate (FDR) correction with α = 0.05 was applied to the 80 regions to correct the *p*-value for multiple comparisons.

Voxel-based morphometry was also used to detect localized differences between the study groups (Figure 1iv). Prior to the Student’s t-tests, the spatially normalized images were smoothed with a Gaussian kernel with a standard deviation that corresponds to the coarsest original image resolution (0.2734 mm in-plane spacing and 1.5 mm slice thickness). Note that a Gaussian kernel standard deviation of two voxels was used in-plane since k-space was zero-filled during image acquisition. Each post-injury image group (P1, P2, P7, and P14) was compared to the baseline image group (B) using two-tailed, unpaired Student’s t-test for detecting statistically significant differences. We used the 3dttest++ program in AFNI (Analysis of Functional NeuroImages, v. 21.3.05) (37) with the -ClustSim option in order to calculate our voxel-based group statistical contrasts and control for multiple comparisons. Note that the use of unpaired t-tests, rather than paired t-tests, was based on the same rationale previously described for the region-based comparisons. The t-value maps were thresholded with an uncorrected *p* < 0.001 to form connected clusters of significantly different voxels. Note that registration errors were inherent due to re-slicing of the anisotropic MR images with large slice thickness (i.e., large slice resolution compared to small in-plane resolution) and slice gaps (i.e., missing volumetric data), requiring use of a smaller significance level, α, during statistical analyses and careful evaluation between the same scans taken in different planes (i.e., axial vs. sagittal). Cluster-level multiple comparisons correction based on Gaussian field theory was carried out within the 3dttest++ *via* the -ClustSim option. After computing each t-test, the 3dttest++ program produced 10,000 randomized permutations of the input data and called the 3dClustSim program in AFNI to approximate the autocorrelation function of the input. The 3dttest++ program was provided the WHS brain mask as additional input in order to limit the t-tests to the brain. Using the tabular output from 3dClustSim, clusters of significant t-values were excluded if smaller than a cutoff size corresponding to a global corrected α = 0.05 and voxel-wise uncorrected *p* < 0.001. Only clusters surviving multiple comparisons correction were reported in the results.

Since we observed vasodilation of the SSS and vasodilation of the SSS has been observed in a similar study (38), we measured the diameter of the SSS in each registered T1W GRE + Gd difference image and compared the change in diameter from B to P14. A custom 2D image viewer graphical user interface programmed in Java was used to select two points of interest within the lumen of the SSS. Relative to bregma in stereotaxic coordinates, these seed points were at offsets [0.0 mm right/left, 1.07 mm ventral, 0.22 mm caudal] and [0.0 mm right/left, 0.53 mm ventral, 5.55 mm caudal]. A sphere was grown from each point until reaching a local maximum image gradient at the boundary of the SSS. The physical diameter of the sphere was used to approximate the diameter of the SSS at these two locations. Each SSS measurement was visually inspected to verify consistent selection of the SSS lumen across all scans. The diameters were compared using one-way ANOVA followed by t-tests for paired group comparisons (α = 0.05).

**Table 6:** Diameter of the SSS in T1W GRE + Gd Diff images and statistical comparisons.

Location vs. Bregma (mm)			Day	n	Mean	SD	SEM	Comparison	Paired Group *t*-tests
RL	VD	RC						Days*	*p*-value*
0.00	−1.07	−0.22	B	18	0.96	0.16	0.04	P1 > B	7.32 × 10^−5^
			P1	10	1.26	0.10	0.03	P1 > P2	3.86 × 10^−2^
			P2	10	1.09	0.17	0.06	P1 > P7	1.36 × 10^−2^
			P7	8	1.06	0.17	0.05	P1 > P14	1.28 × 10^−3^
			P14	9	0.97	0.18	0.06		
0.00	−0.53	−5.55	B	18	0.93	0.17	0.04		
			P1	10	1.17	0.23	0.07		
			P2	8	1.03	0.26	0.09		
			P7	10	1.10	0.25	0.08		
			P14	9	1.01	0.20	0.07		

RL, Right–Left; VD, Ventral-Dorsal; RC, Rostral-Caudal.

* Only comparisons with statistically significant differences are shown (α = 0.05).

## Results

### Region-based comparisons

Region-based statistical comparisons between the baseline and post-injury imaging groups were conducted for each of the 80-segmented regions of the brain in each scan (i.e., T2W FSE, T1W SE, and T1W GRE). Initially, several region-based statistically significant differences were found between groups; however, upon careful inspection of the results by a subject matter expert on quantitative MRI analyses, these differences were determined likely to be false positives (see the Supplemental material for the detailed results and discussion). These false positives were likely caused by the limitations of the data (i.e., image resolution and presence of interslice gaps) presented in detail in the discussion.

### Voxel-based morphometry comparisons

**Figure 2:**
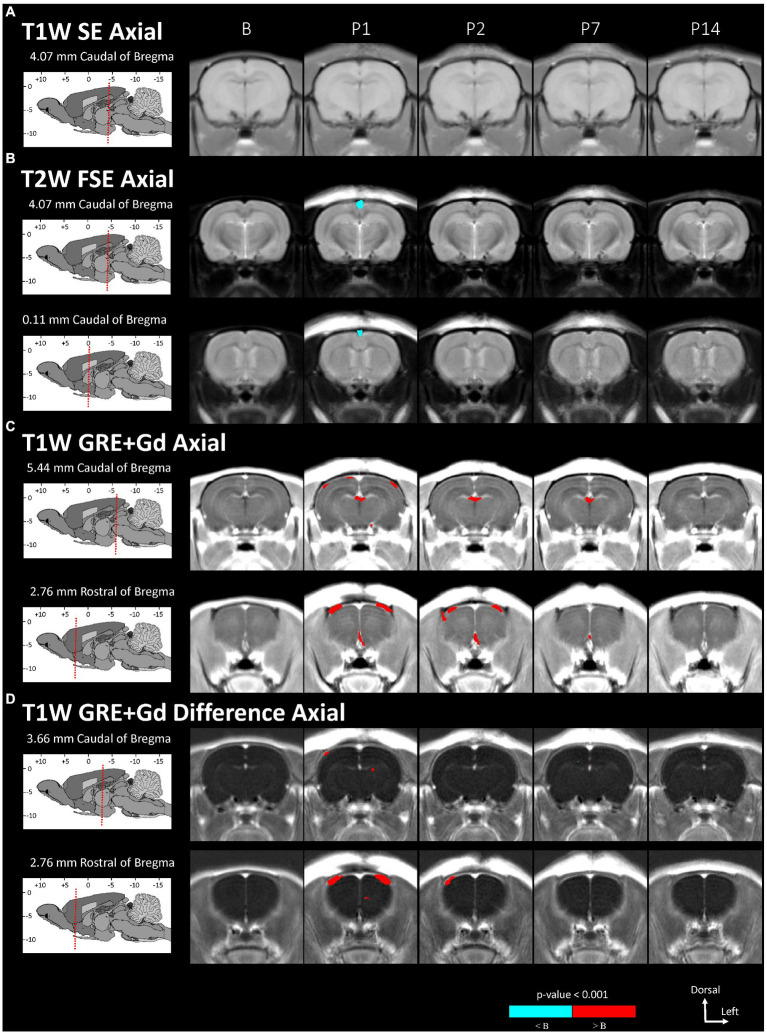
Voxel-based morphometry statistical comparisons between baseline and post-injury images grouped by day and axial scan, showing only the slices with significantly different voxel clusters for each scan **(A-D)**. The grayscale brain MRI underlays shown are the average signals of the images grouped by day.

**Figure 3:**
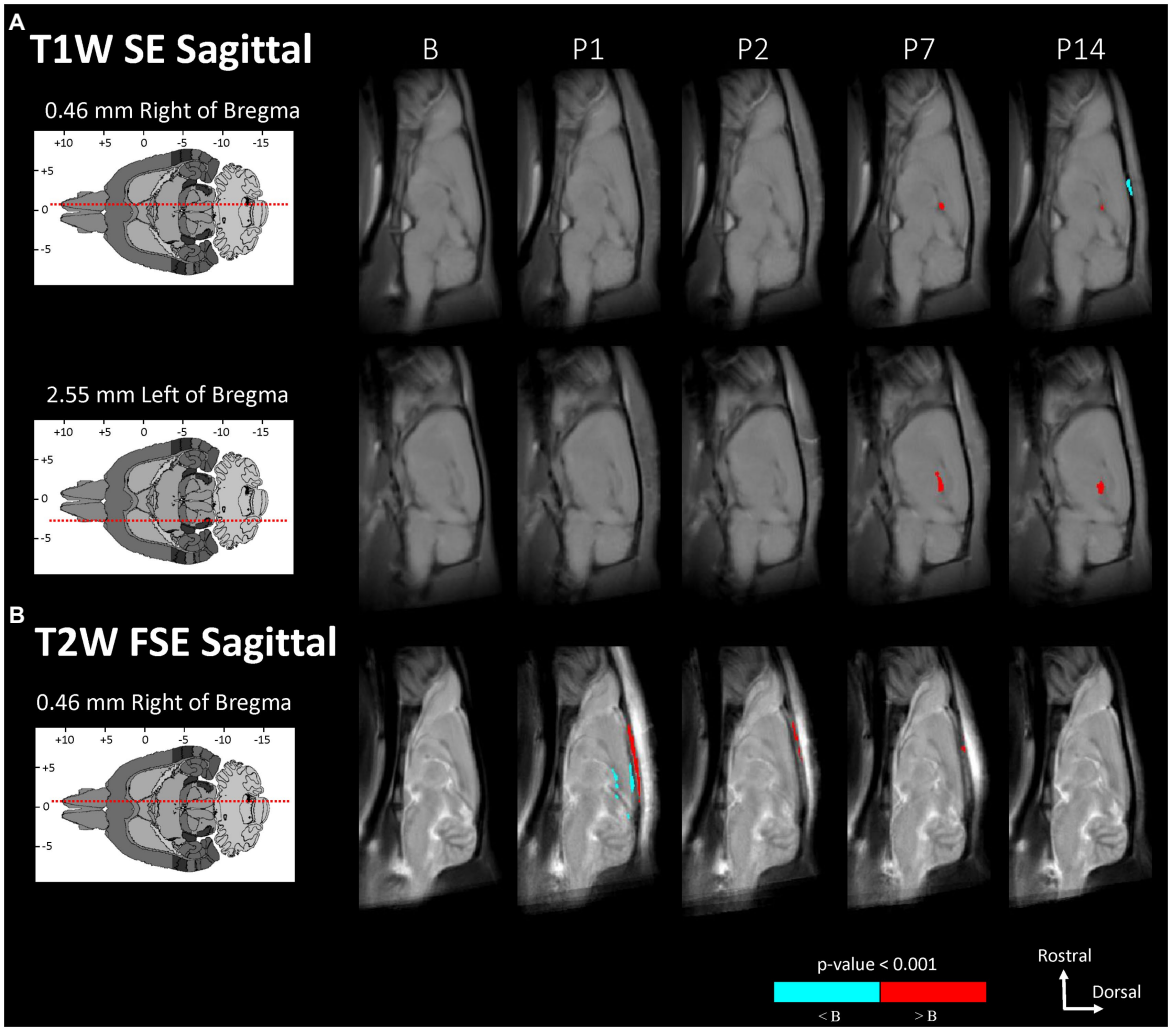
Voxel-based morphometry statistical comparisons between baseline and post-injury images grouped by day and sagittal scan, showing only the slices with significantly different voxel clusters for each scan **(A,B)**. The grayscale brain MRI underlays shown are the average signals of the images grouped by day.

Voxel-based morphometry comparisons revealed localized changes between the baseline and post-injury image groups that were not detected in the region-based comparisons (see Figures 2, 3 and Table 5). In Figures 2, 3, the MRI grayscale underlays of the brain show the group mean intensities and the colored (red and blue) overlays indicating clusters of voxels that were significantly different (*p* < 0.001) from the baseline (B) day; the colored voxels are: red = hyperintensities and blue = hypointensities. The coordinate system insets show the slice position in the WHS atlas in units of mm with bregma as the origin. Only slices with statistically significant findings are shown. Note that unmarked hyperintense areas are clearly evident in all of the images at the site of the surgical incision into the skin and fascia on the superior surface of the cranium (e.g., Figure 2B, P1); this was caused by a combination of surgical damage to the tissues, inflammation, and the presence of skin adhesive for suture. However, these hyperintense areas were outside the brain and were not included in the results since the t-tests for statistically significant differences were performed only within the brain.

**Table 5:** Voxel-based morphometry group statistically significant differences.

Scan type	Image	Cluster size	Maximum	Location vs. Bregma (mm)			Region name
	Comparison	(# Voxels)	t-value	RL	VD	RC	
T1W SE Sagittal	P7 > B	1,518	6.26	3.70	−4.22	−4.87	thalamus
	P14 > B	1,217	5.26	3.16	−4.22	−4.87	thalamus
T2W FSEAxial	P1 > B	3,420	−9.14	0.32	−1.21	−3.11	neocortex
	P7 > B	1,018	−4.85	0.32	−1.07	−4.48	neocortex
T2W FSESagittal	P1 > B	3,096	−4.43	2.20	−3.40	−5.41	cornu ammonis 3
	P1 > B	1,638	−4.83	−0.67	−1.21	−4.87	neocortex
	P1 > B	2,380	4.45	0.69	−0.53	−2.41	neocortex
T1W GRE + GdAxial	P1 > B	1,290	5.82	6.61	−2.44	−3.80	neocortex
	P1 > B	2,434	5.44	5.24	−2.58	1.81	neocortex
	P1 > B	2,986	4.99	−4.33	−1.62	−1.20	neocortex
	P1 > B	1,062	4.57	1.55	−1.35	−1.47	neocortex
	P1 > B	1995	5.90	0.46	−7.50	3.58	olfactory bulb
	P1 > B	848	5.26	1.00	−4.49	−5.71	superior colliculus
	P2 > B	1,641	6.03	0.46	−7.36	1.67	basal forebrain
	P2 > B	1,070	6.22	5.65	−4.22	3.58	neocortex
	P2 > B	1,127	5.48	4.01	−2.30	1.12	neocortex
	P2 > B	622	4.78	−2.96	−2.03	1.67	neocortex
	P2 > B	1,271	5.81	1.14	−4.49	−5.71	pretectal region
	P7 > B	710	5.75	0.46	−7.36	1.40	basal forebrain
	P7 > B	1,087	5.36	1.14	−4.63	−3.52	pretectal region
T1W GRE + GdDifference ImageAxial	P1 > B	934	4.36	5.24	−2.71	2.90	neocortex
	P1 > B	1,162	4.79	6.47	−1.76	−3.52	neocortex
	P1 > B	1,172	5.17	−0.50	−5.58	1.40	striatum
	P1 > B	765	4.62	−1.87	−4.63	−2.57	thalamus
	P2 > B	1,235	4.94	5.24	−2.71	2.90	neocortex
	P7 > B	741	−5.40	2.51	−2.44	−6.39	neocortex
	P7 > B	1,376	4.97	1.28	−0.94	−1.75	neocortex

RL, Right–Left; VD, Ventral-Dorsal; RC, Rostral-Caudal.

Statistically significant hypointensities were found near the mid-sagittal plane near the SSS ventral to the site of impact, on P1 in the axial T2W FSE images (Figure 2B) and in the sagittal T2W FSE images (Figure 3B). In the axial and sagittal T2W FSE images (Figures 2B, 3B), these clusters of hypointensities near the SSS were absent on days P2–14. A similar hypointense cluster was found in the SSS area in both the axial and sagittal T2W FSE images (with a maximum t-value located at bregma offsets of [0.32, −1.21, −3.11] mm and [−0.67, −1.21, −4.87] mm, respectively, which are near each other). This indicates that the cluster is real and not an artifact of the image registration or the anisotropic resolution. However, differences near the SSS were not detected in the axial or sagittal T1W SE images (Figures 2A, 3A).

Referencing diagrams from an anatomical rodent atlas based on MRI-based segmentations of vasculature (39, 40), we compared clusters in the axial T1W GRE + Gd (i.e., Gd-enhanced) and T1W GRE + Gd Difference (i.e., Gd-difference) images with nearby vasculature. Post-injury hyperintensities were found in the Gd-enhanced images (Figure 2C) and the Gd-difference images (Figure 2D) near major vasculature. Hyperintensities in the Gd-enhanced signal (Figure 2C, axial slice: 5.44 mm caudal of bregma) on P1 were present bilaterally on the neocortical surface, near the middle cerebral artery and its branches, and medially on the right neocortical surface, near the SSS. On P1–7, hyperintensities shaped like two vessels were located near the dorsal third ventricle in proximity to the great cerebral vein of Galen and the longitudinal hippocampal vein. At a more rostral slice (Figure 2C, axial slice: 2.76 mm rostral of bregma), hyperintensities were again present bilaterally on the neocortex on days P1–2. Hyperintensities were also located in the basal forebrain region, possibly near the anterior cerebral artery. Both the Gd-enhanced (Figure 2C) and Gd-difference (Figure 2D) images have hyperintense clusters that agree in location (i.e., neocortex) grouped by day (i.e., P1–2).

**Figure 4:**
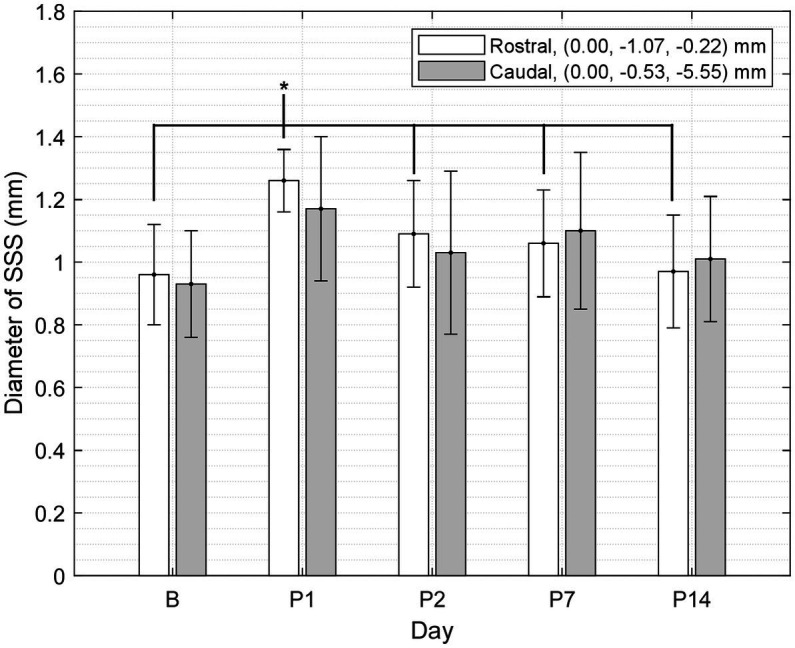
Diameter of the SSS at two points (rostral and caudal) measured in a mid-sagittal slice of the T1W GRE + Gd difference images grouped by day (mean ± SD). At the rostral point, P1 was greater than B and P2–14 (where * indicates *p* < 0.05).

Measurements of the diameter of the SSS from the T1W GRE + Gd difference images showed a statistically significant increase in the diameter of the SSS, i.e., vasodilation of the SSS, following injury at the rostral location of the two points selected within the lumen of the SSS (see Table 6 and Figure 4), whereas no statistically significant increase was found at the caudal location. At the rostral point, there was an acute increase in the diameter of the SSS from B to P1 in all 10 subjects. The diameter then decreased from P1 to P2 in all subjects except one. These measurements also corroborate the voxel-based morphometry that showed a hypointense T2-weighted value near the SSS boundary on P1.

## Discussion

We found no statistically significant changes in the white or gray matter of the brain after mTBI in the region-based analyses of the T2W, T1W, and contrast-enhanced T1W GRE imaging on post-injury days 1, 2, 7, and 14 compared to baseline (see Supplemental information). However, the voxel-based comparisons showed localized, time-dependent, statistically significant differences near vasculature after injury in areas proximal to the SSS, the dorsal third ventricle, and the superior subarachnoid space (SA; Table 6).

The voxel-based comparison results indicate vasodilation or altered blood flow of the cerebrovasculature inferior to site of impact. A hypointense cluster found in the neocortex near the SSS was directly inferior to the site of injury [visible in axial and sagittal T2W images (Figures 2B, 3B)]. Cluster intensity changes proximal to the SSS were most prominent on P1; no difference was seen on P2, P7, or P14. Additionally, measurements of the diameter of the SSS at a rostral cross-section showed a statistically significant increase in the diameter of the SSS on P1 compared to all other days (Figure 4).

Acute dilation of vasculature was also detected more distant from the impact site. Clusters of T1W GRE + Gd signal hyperintensities were observed on P1–7 near the dorsal third ventricle in proximity to the superior colliculus (Figure 2C, 5.44 mm caudal of bregma) and additionally near the medial surface of the basal forebrain (Figure 2C, 2.76 mm rostral of bregma). The Paxinos and Watson stereotaxic atlas (41) at 5.44 mm caudal from the bregma also shows large vessels within the dorsal third ventricle that spatially correspond to our observed clusters of hyperintensities. A detailed atlas of cerebrovasculature prepared by Xiong, Li (40) indicates that vessels near the dorsal third ventricle include the great cerebral vein of Galen and its branches. From the Paxinos and Watson atlas (41) at axial slice 2.76 mm rostral of the bregma, we identified the azygous anterior cerebral artery as a vessel near the observed hyperintense clusters adjacent to the basal forebrain. These hyperintensities within or near the third ventricle appear to be due to adjacent vasculature rather than increased CSF volume, since the segmented CSF regions were not enhanced by the Gd. Hyperintense clusters were also found on the superior surface of the neocortex on P1–2, which appeared as widening of the SA due to dilation of the blood vessels within the SA, in the region directly inferior to the site of impact [visible in the axial T1W GRE Gd-enhanced and difference images (Figures 2C,D) and in the sagittal T2W FSE images (Figure 3B)]. The hyperintensities were most pronounced on P1, with a decline on P2, and a return to baseline on P7.

Although voxel-based differences were detected near known vasculature, no changes were detected in the other deeper tissues of the brain. Furthermore, there were no findings of hemorrhages or BBB dysfunction or disruption. Gd contrast molecules are too large to exit the vasculature of the BBB of normal integrity or permeability and therefore can be used to enhance signals, such as highlighting the cerebral vasculature or BBB, the central nervous system (CNS) tissues that lack a BBB (e.g., pituitary gland), areas of BBB breakdown, and CNS lesions (42). Since we found no statistically significant changes in signal intensities in the deeper brain matter that could be indicative of hemorrhaging or lesions in any of the imaging modalities used in this study, we attribute the influx of the Gd at the superior surface of the neocortex during the first 2 days post-injury to localized vasodilation, possibly caused by inflammation, low tissue oxygenation, or altered blood flow dynamics.

Our results are in agreement with the results found in literature using ≥7 T MRI scanners and show that the 1 T MRI scanner performs at a level comparable to higher field strength scanners used for this type of research. In Braeckman, Descamps (43), mTBI was induced in the rat via the same methods used in this study and *in vivo* T2W imaging of the brain with a 7 T MRI scanner found no abnormalities, including no enlarged ventricles or hemorrhages, on 1 day, 7 days, and 12 weeks post-injury. In a similar study by Hou, Nelson (4), post-mortem histological T2W imaging of rat brain slices at 18 weeks post-injury showed no abnormalities or hemorrhages. In Tu, Williams (44), although rats underwent a modified impact method inducing severe TBI, T2W RARE and T2*W multiple gradient echo imaging via a 7 T MRI scanner showed no abnormalities on 1, 10, 20, and 30 days post-injury compared to baseline. Ventricle dilation was detected in 9 of the 40 TBI subjects on P1 with statistical significance in the T2W images; however, when all subjects were included, there was no significant change in ventricular size over time after TBI. In our study, we found no abnormalities, hemorrhages, or enlarged ventricles in any of our post-injury images. Although we did find hyperintensities near the third dorsal ventricle, the hyperintensities were likely due to dilation of the blood vessels near the ventricle. In another similar study, Shafqat, Christensen (38) found enlarged venous sinuses (i.e., the SSS and left and right transverse sinuses (LTS and RTS)) in T2W Rapid Acquisition with Relaxation Enhancement (RARE) images taken with a 9.4 T MRI scanner. The volume and area of the enlarged brain sinuses were measured and found to be significantly larger on P1 compared to sham, but the size of the sinuses returned to normal on P14; no other changes were found in the images. Shafqat, Christensen (38) used a similar experimental design as our study, but with a modified method and with a much milder impact severity (i.e., a 150 g weight dropped from a height of 0.5 m). The brain sinuses were enlarged due to vasodilation, which was found to be due to CBF alterations and not due to direct impact on the sinus.

The limitations of this study include the use of a 1 T MRI scanner with less spatial resolution, signal-to-noise-ratio (SNR), and modality options as MRI scanners with higher magnetic field strengths used in similar studies. Limitations also include for the T1W SE and T2W FSE images only, highly anisotropic resolution (0.1367 × 0.1367 × 1.5 mm^3^) and interslice gaps (0.5 mm). These protocol parameters were chosen to provide sufficient resolution, SNR, tissue contrast, acquisition time, and whole brain coverage for this study, but resulted in the selection of anisotropic images with interslice gaps. However, the use of anisotropic MRI acquisition for *in vivo* imaging is common, even with >1 T magnetic field scanners (43–46). The combination of anisotropic voxels of the regions and interslice gaps resulted in missing information within the 3D reconstructed brain volume. For example, the highly anisotropic resolution of the images resulted in atlas-defined regions that usually spanned multiple 1.5 mm axial slices, but some of the atlas-defined regions were localized to only one or two sagittal slices in the data. Anisotropy and interslice gaps sometimes led to coincidental differences in comparisons between images taken on different days and were also the primary culprits behind the inconsistent results between the axial and sagittal region-based comparisons, requiring careful interpretation of the results by a subject matter expert. Another limitation is that a sham control group was not used, which makes it difficult to discern the exact mechanism causing vasodilation, i.e., if the vasodilation was caused by the blunt trauma of the drop-weight impact or caused by the surgical procedures needed to perform the drop-weight impact. Since we aimed to investigate if any changes in the brain matter or BBB were quantifiably detectable and statistically significant in the T1W and T2W imaging with a 1 T MRI using this type of mTBI model, we did not include a sham control group. Additionally, the grouped baseline images were used as the negative control group instead of using a separate group of untreated subjects followed out to day 14 out of a concern for animal welfare and reduction.

In order to better understand whether interslice gaps led to bias in the statistical group comparisons, we checked the consistency in subject positioning and compared our statistical image contrasts to the spatial maps of slice-gaps. In the sagittal scans, the center slice was consistently positioned within a small fraction of a millimeter (<100 μm) from the median plane. The central slice positioning in axial scans varied between subjects and days by as much as ±1 mm from the ventral-most point of the cerebral hypophysis. Even with consistent central slice positioning, the anatomical positions of the first and last slice varied by as much as ±1.5 mm due to variability in brain size between subjects. Since our slice positioning varied between scans by as much as a full 1.5 mm slice in some cases, we computed spatial maps of the 0.5 mm slice gaps transformed to the Waxholm atlas space using the same affine transforms as used for spatial normalization. For each image contrast (e.g., T1W SE axial P1 versus B), we viewed the t-value spatial maps side-by-side with the total number of scans with overlapping slice gaps. A striped pattern in slice-gap overlap was observed in all groups, but no such striping pattern was seen in any corresponding t-value map. We conclude from this analysis that group differences were not confounded by spatial variations in the slice gap overlap. It is possible that the variations in positions of slice gaps introduced additional variability and therefore reduced the power of the statistical comparisons.

## Conclusion

The rat animal model was anesthetized and exposed to a model of mTBI via the Marmarou linear acceleration drop-weight technique. Using a low magnetic field, 1 T preclinical scanner to perform structural and contrast-enhanced MRI of the rat brain, we found statistically significant changes in the vasculature of the brain. Voxel-based analyses showed vasodilation or altered blood flow of the SSS and the SA localized to the site of injury, with a visible vasodilation of the SSS on P1 and widening of the superior SA due to vasodilation of the contained blood vessels on P1–2 compared to baseline. Voxel-based analyses also showed possible vasodilation of the vasculature near the dorsal third ventricle and basal forebrain on P1–7 compared to baseline. There were no findings of abnormalities, ventricle enlargement, hemorrhages, or BBB dysfunction or disruption. As a caveat, since we did not perform segmentation of the vasculature or measure vessel cross-sectional areas (because we did not have sufficient resolution or whole brain coverage to do so) and there is no segmentation for vasculature in the WHS rat brain atlas (to our knowledge), our interpretation of the results is limited to visual interpretations of the quantitative comparisons with anatomical diagrams of the vasculature of the rat brain. All the voxel-based, statistically significant changes were found near perivascular areas of the gray and white matter of the injured brain when compared to baseline. These changes were determined not to be directly due to structural neuronal injury in the brain matter, but indirectly due to morphological changes in the vasculature. These observables of mTBI were detectable with a 1 T MRI scanner—a much lower magnetic field strength scanner than used in other previously mentioned studies with similar findings. These findings show that the 1 T MRI scanner used in this study is capable of being used to investigate vascular changes in the brain after mTBI and has the potential to perform a wide range of investigations equivalent to that of higher magnetic field strength MRI scanners.

## Data availability statement

The datasets presented in this article are not readily available because access to the datasets supporting the conclusions of this article may be restricted by the United States Air Force, the Department of Defense, or the United States Government. Requests to access the datasets should be directed to JB, jennie.burns.1@us.af.mil.

## Ethics statement

The animal study was reviewed and approved by Institutional Animal Care and Use Committee (IACUC) at the Air Force Research Laboratory at Joint Base San Antonio (JBSA) Fort Sam Houston, TX.

## Author contributions

JB, CV, and WV: conceptualization and experimental design. JB: MRI scanner setup. JB, MS, CC, and DF: experimental setup, conduction, and data acquisition. DF: dedicated animal care and monitoring. DF and CC: animal post-injury monitoring. JB and BK: data analyses, creation of figures and tables, and writing. All authors contributed to the article and approved the submitted version.

## Funding

The work was supported by the Air Force Research Laboratory, 711th Human Performance Wing, Airman Systems Directorate, Bioeffects Division, Radio Frequency Bioeffects Branch (711 HPW/RHDR).

## Conflict of interest

JB, BK, MS, CC, and DF were employed by General Dynamics Information Technology.

The remaining authors declare that the research was conducted in the absence of any commercial or financial relationships that could be construed as a potential conflict of interest.

## Publisher’s note

All claims expressed in this article are solely those of the authors and do not necessarily represent those of their affiliated organizations, or those of the publisher, the editors and the reviewers. Any product that may be evaluated in this article, or claim that may be made by its manufacturer, is not guaranteed or endorsed by the publisher.

## Figures and Tables

**Table:** 

BBB	Blood–brain barrier
CBF	Cerebral blood flow
CNS	Central nervous system
CSF	Cerebral spinal fluid
Gd	Gadolinium
FDR	False-discovery rate
FOV	Field of view
ITK	Insight tool kit
LTS	Left transverse sinus
MRI	Magnetic resonance imaging
mTBI	Mild traumatic brain injury
NEX	Number of excitations
n_s_	Number of samples
RARE	Rapid acquisition with relaxation enhancement
ROI	Region of interest
RTS	Right transverse sinus
SA	Subarachnoid space
SC	Subcutaneous
SD	Standard deviation
SEM	Standard error of the mean
SNR	Signal-to-noise-ratio
SSS	Superior sagittal sinus
T1W GRE + Gd	T1-weighted gradient echo + gadolinium
T1W 3D GRE	T1-weighted three-dimensional gradient echo
T1W SE	T1-weighted spin echo
T2W FSE	T2-weighted fast spin echo
TBI	Traumatic brain injury
TE	Echo time
TR	Repetition time
WHS	Waxholm space

## Glossary

BBB: Blood–brain barrier
CBF: Cerebral blood flow
CNS: Central nervous system
CSF: Cerebral spinal fluid
Gd: Gadolinium
FDR: False-discovery rate
FOV: Field of view
ITK: Insight tool kit
LTS: Left transverse sinus
MRI: Magnetic resonance imaging
mTBI: Mild traumatic brain injury
NEX: Number of excitations
n_s_: Number of samples
RARE: Rapid acquisition with relaxation enhancement
ROI: Region of interest
RTS: Right transverse sinus
SA: Subarachnoid space
SC: Subcutaneous
SD: Standard deviation
SEM: Standard error of the mean
SNR: Signal-to-noise-ratio
SSS: Superior sagittal sinus
T1W GRE + Gd: T1-weighted gradient echo + gadolinium
T1W 3D GRE: T1-weighted three-dimensional gradient echo
T1W SE: T1-weighted spin echo
T2W FSE: T2-weighted fast spin echo
TBI: Traumatic brain injury
TE: Echo time
TR: Repetition time
WHS: Waxholm space
