# Supplementary material for: Dilation of the superior sagittal sinus detected in rat model of mild traumatic brain injury using 1 T magnetic resonance imaging
Source: Front Neurol. 2023 Apr 26;14:1045695. doi: 10.3389/fneur.2023.1045695 (PMC10169716; doi:10.3389/fneur.2023.1045695)
Supplement: Supplementary file 1 [file Table_1.pdf]

Dilation of the Superior Sagittal Sinus Detected in Rat Model of Mild Traumatic Brain Injury Using 1T Magnetic Resonance Imaging

SUPPLEMENTAL MATERIAL

Region-based Comparisons

Region-based statistical comparisons between the baseline and post-injury imaging groups were conducted for each of the 80-segmented regions of the brain in each scan (i.e., T2W FSE, T1W SE, and T1W GRE). The results, showing erroneous statistically significant differences (i.e., false positives), are presented in Supplementary Table 1. The region-based statistical comparisons of the brain MRIs surprisingly showed quite a few statistically significant differences between the baseline and post-injury images for the 80 segmented regions of the brain evaluated in each scan in the sagittal images of the T1W SE and T2W FSE scans. The T1W SE signal in regions near the sagittal center of the brain was significantly greater post-injury in the sagittal scans. T1W SE hyperintensities on days P1, P2, and P14 were detected in the ventricular system and superior colliculus. The only region with significant decreases in the mean T1W SE signal was the neocortex on P2 and P14. Significant decreases in the mean T2W FSE signal were detected in the sagittal scans on P1 in multiple hippocampal areas that include the cornu ammonis 1, cornu ammonis 2, dentate gyrus, subiculum, and fasciola cinereum. On P2, a significant decrease in the mean T2W FSE signal was found in the commissure of the inferior colliculus. No significant differences in the regional mean signal were detected in the axial T2W FSE, T1W SE, or T1W GRE+Gd scans. However, note that all region-based differences were detected in sagittal scans, but not in their axial counterparts. We suspect that finding no regional differences in their axial counterparts may be due to the aforementioned limitations of the data, i.e., image resolution and presence of interslice gaps (see the section, “Limitations,” in the paper for more detail). Therefore, any statistically significant region-based differences found between groups (Supplementary Table 1) were likely false positives. We showed and discussed this here for explanatory purposes.

Supplementary Table 1. Region-based statistically significant differences between groups; however; the differences are likely false positives. (All uncorrected p-values are shown that survived FDR correction with alpha = 0.05).

Scan Type	Image Comparison		t-value	p-value	Region Name
T1W SE Sagittal	P1>B		4.66	3.6E-5	brachium of the superior colliculus
	P1>B		2.92	3.4E-3	fasciola cinereum
	P1>B		3.51	7.6E-4	habenular commissure
	P1>B		3.27	1.4E-3	stria medullaris of the thalamus
	P1>B		3.19	1.8E-3	superficial gray layer superior colliculus
	P1>B		4.46	6.1E-5	ventricular system
	P2>B		4.25	1.1E-4	brachium of the superior colliculus
	P2>B		2.86	3.9E-3	commissure of the superior colliculus
	P2>B		3.35	1.2E-3	deeper layers of the superior colliculus
	P2>B		2.78	4.8E-3	dentate gyrus
	P2>B		4.39	7.2E-5	habenular commissure
	P2>B		3.89	2.8E-4	inferior colliculus
	P2>B		2.82	4.4E-3	molecular layer of the cerebellum
	P2>B		-2.77	4.9E-3	neocortex
	P2>B		3.78	3.8E-4	periventricular gray
	P2>B		3.58	6.4E-4	pretectal region
	P2>B		4.61	4.0E-5	stria medullaris of the thalamus
	P2>B		3.42	9.8E-4	superficial gray layer superior colliculus
	P2>B		3.05	2.5E-3	thalamus
	P2>B		3.15	1.9E-3	ventricular system
	P7>B		3.68	4.9E-4	brachium of the superior colliculus
	P14>B		6.12	1.0E-6	brachium of the superior colliculus
	P14>B		3.14	2.0E-3	deeper layers of the superior colliculus
	P14>B		3.42	9.7E-4	dentate gyrus
	P14>B		3.42	9.6E-4	habenular commissure
	P14>B		2.93	3.4E-3	inferior colliculus
	P14>B		-2.74	5.3E-3	neocortex
	P14>B		2.71	5.7E-3	presubiculum
	P14>B		3.16	1.9E-3	pretectal region
	P14>B		4.18	1.3E-4	stria medullaris of the thalamus
	P14>B		3.63	5.7E-4	superficial gray layer superior colliculus
	P14>B		4.37	7.7E-5	ventricular system
T2W FSE Sagittal	P1>B		-4.17	1.3E-4	cornu ammonis 1
	P1>B		-3.48	8.2E-4	cornu ammonis 2
	P1>B		-3.08	2.3E-3	dentate gyrus
	P1>B		-3.94	2.4E-4	fasciola cinereum
	P1>B		-3.09	2.2E-3	subiculum
	P2>B		-4.21	1.2E-4	commissure of the inferior colliculus
